# Supplementary material for: Association between local food policy council coverage and longitudinal household food insufficiency during COVID-19, stratified by race, ethnicity, and income
Source: PLoS One. 2026 Mar 25;21(3):e0345654. doi: 10.1371/journal.pone.0345654 (PMC13016277; doi:10.1371/journal.pone.0345654)
Supplement: S1 Table — (DOCX) [file pone.0345654.s001.docx]

**S1Table.** U.S. Census Household Pulse Survey data collection cycle dates and corresponding time point in an analysis of household food insufficiency during the COVID-19 public health from May 2020 to 20203.

| Week | Survey Database Cycle | Cycle End Date | Time Point in analysis |
| --- | --- | --- | --- |
| 1 | Household Pulse Survey PUF: April 23 – May 5 | May 5, 2020 | May 2020 |
| 2 | Household Pulse Survey PUF: May 7 – May 12 | May 12, 2020 | May 2020 |
| 3 | Household Pulse Survey PUF: May 14 – May 19 | May 19, 2020 | May 2020 |
| 4 | Household Pulse Survey PUF: May 21 – May 26 | May 26, 2020 | May 2020 |
| 5 | Household Pulse Survey PUF: May 28 – June 2 | June 2, 2020 | June 2020 |
| 6 | Household Pulse Survey PUF: June 4 – June 9 | June 9, 2020 | June 2020 |
| 7 | Household Pulse Survey PUF: June 11 – June 16 | June 16, 2020 | June 2020 |
| 8 | Household Pulse Survey PUF: June 18 – June 23 | June 23, 2020 | June 2020 |
| 9 | Household Pulse Survey PUF: June 25 – June 30 | June 30, 2020 | June 2020 |
| 10 | Household Pulse Survey PUF: July 2 – July 7 | July 7, 2020 | July 2020 |
| 11 | Household Pulse Survey PUF: July 9 – July 14 | July 14, 2020 | July 2020 |
| 12 | Household Pulse Survey PUF: July 16 – July 21 | July 21, 2020 | July 2020 |
| 13 | Household Pulse Survey PUF: August 19 – August 31 | August 31, 2020 | August 2020 |
| 14 | Household Pulse Survey PUF: September 2 – September 14 | September 14, 2020 | September 2020 |
| 15 | Household Pulse Survey PUF: September 16 – September 28 | September 28, 2020 | September 2020 |
| 16 | Household Pulse Survey PUF: September 30 – October 12 | October 12, 2020 | October 2020 |
| 17 | Household Pulse Survey PUF: October 14 – October 26 | October 26, 2020 | October 2020 |
| 18 | Household Pulse Survey PUF: October 28 – November 9 | November 9, 2020 | November 2020 |
| 19 | Household Pulse Survey PUF: November 11 – November 23 | November 23, 2020 | November 2020 |
| 20 | Household Pulse Survey PUF: November 25 – December 7 | December 7, 2020 | December 2020 |
| 21 | Household Pulse Survey PUF: December 9 – December 21 | December 21, 2020 | December 2020 |
| 22 | Household Pulse Survey PUF: January 6 – January 18 | January 18, 2021 | January 2021 |
| 23 | Household Pulse Survey PUF: January 20 – February 1 | February 1, 2021 | February 2021 |
| 24 | Household Pulse Survey PUF: February 3 – February 15 | February 15, 2021 | February 2021 |
| 25 | Household Pulse Survey PUF: February 17 – March 1 | March 1, 2021 | March 2021 |
| 26 | Household Pulse Survey PUF: March 3 – March 15 | March 15, 2021 | March 2021 |
| 27 | Household Pulse Survey PUF: March 17 – March 29 | March 29, 2021 | March 2021 |
| 28 | Household Pulse Survey PUF: April 14 – April 26 | April 26, 2021 | April 2021 |
| 29 | Household Pulse Survey PUF: April 28 – May 10 | May 10, 2021 | May 2021 |
| 30 | Household Pulse Survey PUF: May 12 – May 24 | May 24, 2021 | May 2021 |
| 31 | Household Pulse Survey PUF: May 26 – June 7 | June 7, 2021 | June 2021 |
| 32 | Household Pulse Survey PUF: June 9 – June 21 | June 21, 2021 | June 2021 |
| 33 | Household Pulse Survey PUF: June 23 – July 5 | July 5, 2021 | July 2021 |
| 34 | Household Pulse Survey PUF: July 21 – August 2 | August 2, 2021 | August 2021 |
| 35 | Household Pulse Survey PUF: August 4 – August 16 | August 16, 2021 | August 2021 |
| 36 | Household Pulse Survey PUF: August 18 – August 30 | August 30, 2021 | August 2021 |
| 37 | Household Pulse Survey PUF: September 1 – September 13 | September 13, 2021 | September 2021 |
| 38 | Household Pulse Survey PUF: September 15 – September 27 | September 27, 2021 | September 2021 |
| 39 | Household Pulse Survey PUF: September 29 – October 11 | October 11, 2021 | October 2021 |
| 40 | Household Pulse Survey PUF: December 1 – December 13 | December 13, 2021 | December 2021 |
| 41 | Household Pulse Survey PUF: December 29 – January 10 | January 10, 2022 | January 2022 |
| 42 | Household Pulse Survey PUF: January 26 – February 7 | February 7, 2022 | February 2022 |
| 43 | Household Pulse Survey PUF: March 2 – March 14 | March 14, 2022 | March 2022 |
| 44 | Household Pulse Survey PUF: March 30 – April 11 | April 11, 2022 | April 2022 |
| 45 | Household Pulse Survey PUF: April 27 – May 9 | May 9, 2022 | May 2022 |
| 46 | Household Pulse Survey PUF: June 1 – June 13 | June 13, 2022 | June 2022 |
| 47 | Household Pulse Survey PUF: June 29 – July 11 | July 11, 2022 | July 2022 |
| 48 | Household Pulse Survey PUF: July 27 – August 8 | August 8, 2022 | August 2022 |
| 49 | Household Pulse Survey PUF: September 14 – September 28 | September 28, 2022 | September 2022 |
| 50 | Household Pulse Survey PUF: October 5 – October 17 | October 17, 2022 | October 2022 |
| 51 | Household Pulse Survey PUF: November 2 – November 14 | November 14, 2022 | November 2022 |
| 52 | Household Pulse Survey PUF: December 9 – December 19 | December 19, 2022 | December 2022 |
| 53 | Household Pulse Survey PUF: January 4 – January 16 | January 16, 2023 | January 2023 |
| 54 | Household Pulse Survey PUF: February 1 – February 13 | February 13, 2023 | February 2023 |
| 55 | Household Pulse Survey PUF: March 1 – March 13 | March 13, 2023 | March 2023 |
| 56 | Household Pulse Survey PUF: March 29 – April 10 | April 10, 2023 | April 2023 |
| 57 | Household Pulse Survey PUF: April 26 – May 8 | May 8, 2023 | May 2023 |
