# Supplementary material for: Association between local food policy council coverage and longitudinal household food insufficiency during COVID-19, stratified by race, ethnicity, and income
Source: PLoS One. 2026 Mar 25;21(3):e0345654. doi: 10.1371/journal.pone.0345654 (PMC13016277; doi:10.1371/journal.pone.0345654)
Supplement: S2 Table — (DOCX) [file pone.0345654.s002.docx]

**S2 Table.** Characteristics comparison between analytical and exclusion sample of Census Household Pulse Survey respondents included in an analysis of food sufficiency during the COVID-19 pandemic (March 2020 – May 2023) by food policy council coverage.

| **Individual Level Variables** | **Analytical Sample (N = 3,500,436)** | **Exclusion of No Income Record (N = 518,227)** |
| --- | --- | --- |
| Age in years, mean (sd) | 53.8 (15.6) | 53.0 (17.0) |
| Gender, n (%) |  |  |
| Male | 1445300 (41.3%) | 206763 (39.9%) |
| Female | 2055136 (58.7%) | 311464 (60.1%) |
| Ethnicity and race, n (%) |  |  |
| Hispanic | 292178 (8.3%) | 56950 (11.0%) |
| White, non-Hispanic | 2694611 (77.0%) | 366073 (70.6%) |
| Black, non-Hispanic | 229051 (6.5%) | 48422 (9.4%) |
| Asian, non-Hispanic | 157404 (4.5%) | 26879 (5.2%) |
| Other, non-Hispanic | 127192 (3.7%) | 19903 (3.8%) |
| Education level, n (%) |  |  |
| Less than high school | 59730 (1.7%) | 16970 (3.3%) |
| High school or equivalent | 379633 (10.8%) | 78210 (15.1%) |
| More than high school | 3061073 (87.5%) | 423047 (81.6%) |
| Number of non-adults, n (%) |  |  |
| 0 | 2347412 (67.1%) | 315173 (60.8%) |
| 1 or 2 | 924270 (26.4%) | 160398 (31.0%) |
| 3 or more | 228754 (6.5%) | 42656 (8.2%) |
