# Supplementary material for: Association between local food policy council coverage and longitudinal household food insufficiency during COVID-19, stratified by race, ethnicity, and income
Source: PLoS One. 2026 Mar 25;21(3):e0345654. doi: 10.1371/journal.pone.0345654 (PMC13016277; doi:10.1371/journal.pone.0345654)
Supplement: S1 Fig — (a) in all households, (b) Asian non-Hispanic households, (c) Black non-Hispanic households, (d) white non-Hispanic households, (e) Hispanic households, and (f) other non-Hispanic households. Dotted line represents prevalence ratio of 1.0 (i.e., no difference between food insufficiency prevalence among households in states with low compared to high active local food policy council coverage). (DOCX) [file pone.0345654.s003.docx]

**S1 Fig**. Quarterly prevalence ratios and 95% confidence intervals (95% CIs) of food insufficiency reported on the US Census Household Pulse Survey between May 2020 and May 2023 comparing states with low (<15%) and high (>15%) active local food policy council coverage by race and ethnic-specific household income tertiles (a) in all households, (b) Asian non-Hispanic households, (c) Black non-Hispanic households, (d) white non-Hispanic households, (e) Hispanic households, and (f) other non-Hispanic households. Dotted line represents prevalence ratio of 1.0 (i.e., no difference between food insufficiency prevalence among households in states with low compared to high active local food policy council coverage).

b) Asian households

a) All households

d) White, non-Hispanic households

c) Black, non-Hispanic households

f) Other, non-Hispanic households

e) Hispanic households
