# Supplementary material for: Association between local food policy council coverage and longitudinal household food insufficiency during COVID-19, stratified by race, ethnicity, and income
Source: PLoS One. 2026 Mar 25;21(3):e0345654. doi: 10.1371/journal.pone.0345654 (PMC13016277; doi:10.1371/journal.pone.0345654)
Supplement: Attachment — Submitted filename: Response to Reviewers_FINAL.docx [file pone.0345654.s005.docx]

FPC paper – Response to reviewers

**Editor comments:**

1.Please ensure that your manuscript meets PLOS ONE's style requirements, including those for file naming. The PLOS ONE style templates can be found at

<https://journals.plos.org/plosone/s/file?id=wjVg/PLOSOne_formatting_sample_main_body.pdf> and

[https://journals.plos.org/plosone/s/file?id=ba62/PLOSOne_formatting_sample_title_authors_affiliations.pdf](https://track.editorialmanager.com/CL0/https:%2F%2Fjournals.plos.org%2Fplosone%2Fs%2Ffile%3Fid=ba62%2FPLOSOne_formatting_sample_title_authors_affiliations.pdf/1/010f019a94f0673d-a27f7141-cecc-4eca-8160-e0ceb693e91e-000000/AhS-vXcqNu8xEvtLmRGmEHNJ8DvWGdl8Sa_MrS12RiA=237)

*Thank you, the revised manuscript adheres to PLOS ONE’s style requirements now.*

2. Please state what role the funders took in the study.  If the funders had no role, please state: "The funders had no role in study design, data collection and analysis, decision to publish, or preparation of the manuscript."

*Thank you, we included the statement above after our financial disclosures.*

3. Thank you for uploading your study's underlying data set. Unfortunately, the repository you have noted in your Data Availability statement does not qualify as an acceptable data repository according to PLOS's standards.

At this time, please upload the minimal data set necessary to replicate your study's findings to a stable, public repository (such as figshare or Dryad) and provide us with the relevant URLs, DOIs, or accession numbers that may be used to access these data. For a list of recommended repositories and additional information on PLOS standards for data deposition, please see [https://journals.plos.org/plosone/s/recommended-repositories](https://track.editorialmanager.com/CL0/https:%2F%2Fjournals.plos.org%2Fplosone%2Fs%2Frecommended-repositories/1/010f019a94f0673d-a27f7141-cecc-4eca-8160-e0ceb693e91e-000000/6SwZ-AAPCkLvuGUr70JLJI6VFjTe15oHMPuaZ9Tjn0Q=237).

*We checked the recommended repositories and Harvard Dataverse Network is on the list of recommended options. Our data is published there. I used the Harvard Dataverse Network link from the PLOS ONE link pasted above and searched our study to double check and indeed, it is all our data is available on that repository. We provided the relevant DOI and related data information.*

4. We note that Figure 1 in your submission contain map images which may be copyrighted.

*Thank you, we created a new figure using ArcGIS and acknowledged the data source documentation on the figure and in the methods section (“All spatial processing and map visualizations were conducted in ArcGIS Pro (version 3.5.3, Esri Inc, 2025, Redlands, CA). US state boundary shapefiles were obtained from the US Census Bureau’s TIGER/Line dataset.)”*

5. Please include captions for your Supporting Information files at the end of your manuscript, and update any in-text citations to match accordingly. Please see our Supporting Information guidelines for more information: <http://journals.plos.org/plosone/s/supporting-information>.

*We added captions for supplemental files to the end of the manuscript and followed supplemental material naming conventions.*

6. If the reviewer comments include a recommendation to cite specific previously published works, please review and evaluate these publications to determine whether they are relevant and should be cited. There is no requirement to cite these works unless the editor has indicated otherwise.

*The reviewers did not recommend citing specific previously published works.*

**Reviewer #1**

Abstract
1. Well written and follows the guidelines

*Thank you*

Introduction
1. Overall, well written and describes the rationale behind the potential association of FPC coverage and food insufficiency as well as using the COVID-19 pandemic as a natural experiment.

*Thank you*

Methods
1. Line 140, change percent to percentage

*Done*

2. FPC coverage is based on 2021 data but outcome data spans a wider range, it may be appropriate to use FPC coverage for each year

*The Food Policy Network surveys FPCs every 2 years and there are few changes in the number of councils each year. There is also a delay between when the survey is fielded and when the results are available. Therefore, we are confident that the 2021 data is a strong (and feasible) representation of FPC coverage across the food insufficiency data range.*

3. FPC coverage is dichotomous at the level of 15% coverage, with the explanation that this splits the number of states in half. Is this method appropriate given the wide range of FPC coverage within each group? (0-15% and 16%+) Would it be more appropriate to analyze this data using a continuous model?

*Council coverage was dichotomized at 15% to ease the interpretation of the regression results as this factor was included in the model in a three-way cross-product term along with quarter and income level. Had council coverage been analyzed as a continuous variable, the results would have been reported as regression beta coefficients rather prevalence and prevalence ratios, which we believe are more meaningful.*

4. Income is organized into tertiles, but not based on income levels by state; cost of living varies greatly across this geographic distribution so it may be more appropriate to organize these data with respect to state level income distributions

*We agree that cost of living varies from state to state as well as many other factors that affect a person’s socioeconomic status, including within-state cost of living variation and racial/ethnic identity. Early on in our analyses we recognized that the dollar value corresponding to the lower, middle, and upper tertile of household income varied by racial/ethnic identity and therefore different tertile cut points were assigned to recognize the income disparity.*

5. A high number of participants are excluded due to no record of income. This should be commented on in greater detail. This excluded group may represent a distinct subset of the total population (such as low income or minority race/ethnicity).

*We compared the excluded group to the included group and reported the findings in a new table (Suppl Table 2). We summarized the differences in the results section (“More Hispanic, Black, and Asian households were excluded due to missing household income data compared to white households and more households with high school or less education were excluded than households with higher education levels) and commented on the differences in the limitations section of the discussion (“Households that were excluded due to missing household income data were more likely to be racial or ethnic minorities and report lower levels of education; such factors are associated with higher risk of food insufficiency, so we may have underestimated food insufficiency in our analysis.”)*

Results
1. Many of the results are reported as statistically significant but represent a numerically small difference. The authors could comment on the practical significance of the difference, especially with respect to policy implications

*Good point. We revised the sentence reporting our main result to read: “Overall, slightly fewer households in states where more than 15% of their population lived in an area with an active FPC reported food insufficiency during the COVID-19 pandemic than households in states with lower FPC coverage according to multivariate regression modeling.”*

*We also added “small” to this sentence: “High FPC coverage was associated with small, significantly lower prevalence of food insufficiency during the pandemic among non-Hispanic Black and white households in the lower third income categories compared to similar households in states with low FPC coverage (****Figure 2c,d****).”*

*We also added a sentence to the end of the paragraph about policy supports during the pandemic that reads: “More research is needed to determine whether pandemic relief policy supports diminished or amplified the association between FPC coverage and household food insufficiency.”*

Discussion
1. There is a high percentage of greater than high school education in this data, this should be commented on in more detail

*Good point. We added the following sentences to the limitations paragraph in the discussion: “Our sample contained a higher percentage of greater than a high school degree than the national average (85% in our sample compared to 63.2% nationally (40). Completing at least some college is associated with less food insecurity after controlling for poverty, suggesting our sample may be less susceptible to food insufficiency than the general population (41).”*

2. Conclusions should be described as an association instead of a potential effect or causal factor.

*We modified the first sentence of the conclusion to read: “A higher prevalence of local FPCs in a state was associated with lower prevalence of household food insufficiency during the COVID-19 pandemic among low-income and minoritized households.”*

3. More discussion should be dedicated to state level confounders including heterogenous state-level COVID response.

*Thank you, we added the following sentences to the limitations paragraph in the discussion: “For example, SNAP recipients in some states could use their benefits to purchase groceries online by May 2020 while online shopping implementation took much longer in other states (43). We could not account for the broad heterogeneity in how states responded to food insufficiency during the pandemic in this analysis.”*

**Reviewer #2**:

Lines 68-71 This sentence is structurally difficult to read. I think if the in parenthesis examples were removed from their current placement and included at the end of the sentence that it would be clearer for the reader. "..for similar and policy specific reasons such as (insert examples)."

*Thank you, we revised that sentence based on your recommendation.*

Line 237 This sentence seems to contradict itself with higher percentage in lower coverage. The text immediate preceding the percentage in parentheses says the percentage was lower in the low coverage states.

*Thank you, we changed that sentence to read: “Individual and state level characteristics were similar in both groups of states.”*

Line 291-295 Please consider breaking into two separate sentences for easier readability.

*We broke it into two sentences.*
